# Bibliometric analysis of studies on the lipid management of coronary heart disease from 2014 to 2023

**DOI:** 10.3389/fcvm.2025.1513576

**Published:** 2026-02-02

**Authors:** Si-Qi Liu, Xin-Yu Ji, Shu-Han Zhao, Hai-Yi Liang, Fu-Yi Yang, Yuan-Hui Hu, Shuai Shi

**Affiliations:** 1 Department of Cardiovascular Diseases, Guang'anmen Hospital, China Academy of Chinese Medical Sciences, Beijing, China; 2 Graduate School, Beijing University of Chinese Medicine, Beijing, China; 3 Institute of Basic Research in Clinical Medicine, China Academy of Chinese Medical Sciences, Beijing, China; 4 Third Affiliated Hospital, Beijing University of Chinese Medicine, Beijing, China

**Keywords:** coronary heart disease, lipid management, bibliometrics, Web of Science, risk factors

## Abstract

**Objective:**

The incidence of coronary heart disease (CHD) is progressively increasing on an annual basis. Dyslipidemia constitutes a significant pathogenic factor in CHD, exerting a substantial influence on its onset and progression. Consequently, precise and effective lipid management is crucial for the prevention and cure of CHD. This study aims to examine the risk factors, therapeutic approaches, current research status and emerging trends in lipid management related to CHD.

**Method:**

We searched for publications on lipid management of coronary heart disease from 01/01/2014 to 12/31/2023 in Web of Science and performed bibliometrics using CiteSpace, VOSviewer, Scimago Graphica, Gephi and R Studio.

**Results:**

A total of 6,027 related articles were retrieved from the Web of Science database. After screening, 5,954 articles were included. Journal of Clinical Lipidology and Atherosclerosis were the journals with the most publications and citations, respectively. In this discipline, the United States has the largest number of publications, research institutions, citations, and collaborative partnerships. The burst keywords include sex difference, heterozygote familial hypercholesterolemia, PCSK9, lipid peroxidation, fish oil, monoclonal antibody, insulin sensitivity, and gene, etc.

**Conclusion:**

Research on CHD indicates that risk factors influencing lipid levels encompass sex, genetics, PCSK9, and lipid peroxidation. There has been a growing trend in investigating the underlying mechanisms of these factors. Recent research hotspots have concentrated on disease prevention, prognosis, specific treatments for CHD, the development of new pharmaceuticals, and the molecular mechanisms of action. Future research is likely to continue focusing on more precise treatment protocols and the exploration of novel mechanisms.

## 1 Introduction

Currently, there are 523 million cases of cardiovascular disease (CVD) worldwide, accounting for 6.5% of the world's population (1), which has had a detrimental effect on the economy, society and health. The most frequent cause of CVD death is coronary atherosclerosis, which accounts for 44% of deaths from CVD in men and 38% in women (2).One significant pathogenic mechanism of atherosclerosis is lipid infiltration. As a prevalent form of ischemic heart disease, accurate and effective lipid management is crucial for the prevention.

Precise and efficient lipid management can stabilize the plaque in the arterial wall, stop plaque rupture and thrombosis, slow down the progression of CHD and lower the risk of an acute myocardial infarction. Statins are the most widely used drug therapy. In the treatment of CHD, LDL-C is the key target. Various LDL-C levels should be used to differentiate the disease state, and appropriate interventions should be implemented. In the case of unsatisfactory lipid lowering, intensive treatment or combined treatment is required. Rich in Omega 3 fatty acids, fish oil can lower triglyceride and cholesterol levels, help to reduce the risk of cardiovascular disease. Fish oil is therefore frequently added to the diet of CHD patients as an auxiliary therapy. In non-drug therapy, normal lipid levels are primarily restored by means of dietary modifications, lifestyle adjustments, motion, acupuncture, acupoint sticking, massage, cupping, and other techniques (3–6), all of which are beneficial in both the prevention and treatment of congestive heart failure.

Despite the fact that lipid management is entwined with prevention and therapy on CHD, no bibliometric study exists to evaluate pertinent literature and enumerate research hotspots and evolving trends in this area. In order to better understand the relationship between CHD and lipid management, this paper will analyze pertinent literature through visualization, investigate potential mechanisms of interaction, and examine development trends in this field. We select literature from the Web of Science, integrate and analyze the results using the literature metrology approach, through which includes the most popular research topics, keywords, lead authors, scientific organizations, nations, and journals. Therefore, we further analyze and summarize the influencing factors, research hotspots and development trends of lipid management of CHD, so as to provide reference for clinical research and practice.

## 2 Materials and methods

**Figure 1:**
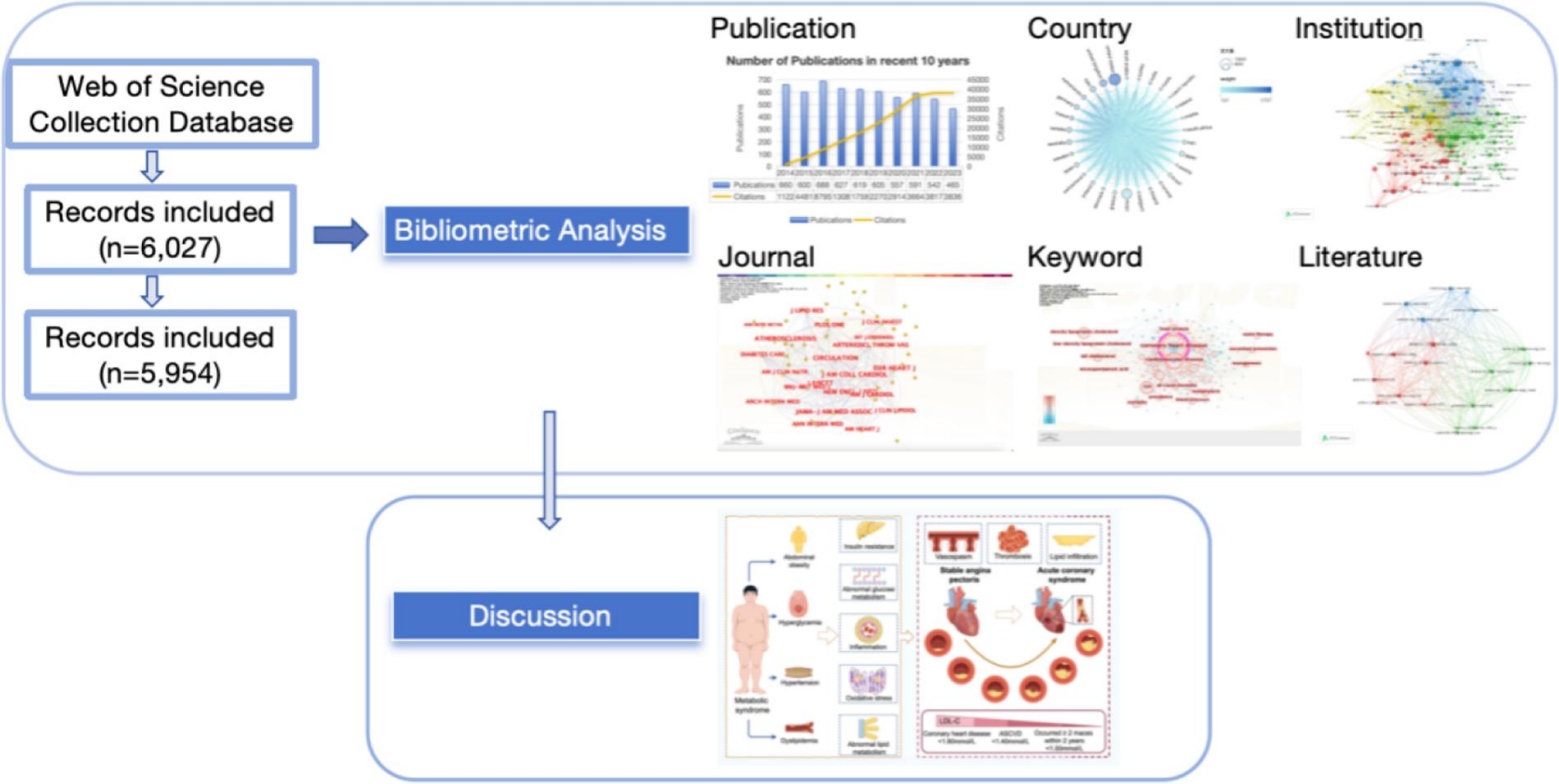
Flow diagram of the literature.

Science mapping is an essential procedure in bibliometrics. It can represent the discipline situation and development status. We use CiteSpace, VOSviewer, Scimago Graphica and Gephi to make visualization mappings in this paper. We conducted a comprehensive search of relevant literature data in Web of Science using the following search strategy: ((([TS = (lipid management)] OR TS = (lipid lowering)) OR TS = (lipid metabolism)) OR TS = (lipid reducing)) AND TS = (coronary heart disease) from January 1, 2014, to December 31, 2023. In order to avoid the error caused by the technical update of the website, all searches and screening were completed in June 1, 2024. A total of 6,026 articles met the requirements. In the document types, we chose “article” and “review article” to ensure the quality of the selected literature. Two people screened and checked the literature back-to-back, and a third person checked the contentious or disputed parts. The search was conducted with the option “All records and references cited” and exported as “download_XX.txt” files. After preprocessing, 5,954 records were included in the analysis. The process of this article is illustrated in Figure 1. In this study, CiteSpace(6.1.R6) (7) and VOSviewer(version 1.6.17) (8) are used as tools to perform the co-citation analysis to identify research hotspots and predict development trends. CiteSpace can be used to perform basic analyses of literature, such as analyzing the number of citations and publications, key journals, author analysis, research and collaborations in institutions, clustering and bursting keyword analysis. In this paper, we mainly apply CiteSpace (6.3.1) software for journal analysis, cluster analysis and bursting keyword analysis. The co-citation analysis of the literature shows the number of publications in scientific journals, the intensity of collaborations and the main years of publication. Cluster analysis clusters the keywords by an algorithm to get the keywords represented by each cluster and assigns a label to each cluster. The more keywords in the clusters, the more significant the cluster structure is represented. Selecting LLR clustering method and clicking “Find Clusters” to explore the common themes of similar documents can help us discover the hidden patterns and regularities in a large amount of text data. Click “Burstness” in the control panel to extract and detect burst terms, and click “Refresh” to calculate the number of bursting keywords. Adjust the Y value to get the suitable number of burst terms, and select the top 25 to be analyzed, in order to understand the frontiers of research, the shift of research focus and the latest research hotspot dynamics, and to help predict the subsequent development trend of the field.

VOSviewer is used to analyze institutions, countries, journals and keywords. The results were shown in different forms, including network visualization, overlay visualization and density visualization to reflect different emphases. Scimago Graphica (9) and Gephi (10) are used for mapping of country co-operation and keyword time zones.

In the clustering maps made by CiteSpace and the analysis of countries made by VOSviewer, colors indicate time. The darker the color, the earlier the time; the lighter the color, the more recent the time. The density visualization map is the same as the notes of cluster maps.

## 3 Results

### 3.1 Distribution of annual publications

**Figure 2:**
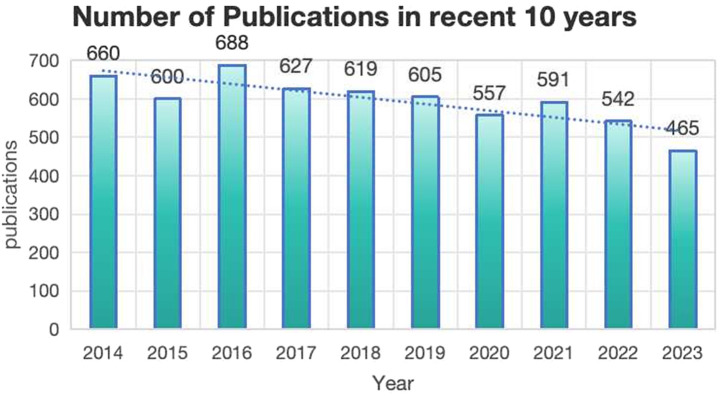
Total number of publications on CHD and lipid management research.

Figure 2 displays the publishing trend from 2014 to 2023 in this field. From 2014 (660) to 2023 (465), the overall number of publications showed a slight downward trend. However, according to the data form WOS, the number of citations showed an upward trend. It is a reflection of the growing understanding of lipid management in CHD. Researchers are becoming more and more interested in this subject of study as it has reached a certain scale.

### 3.2 Author co-citation analysis

**Figure 3:**
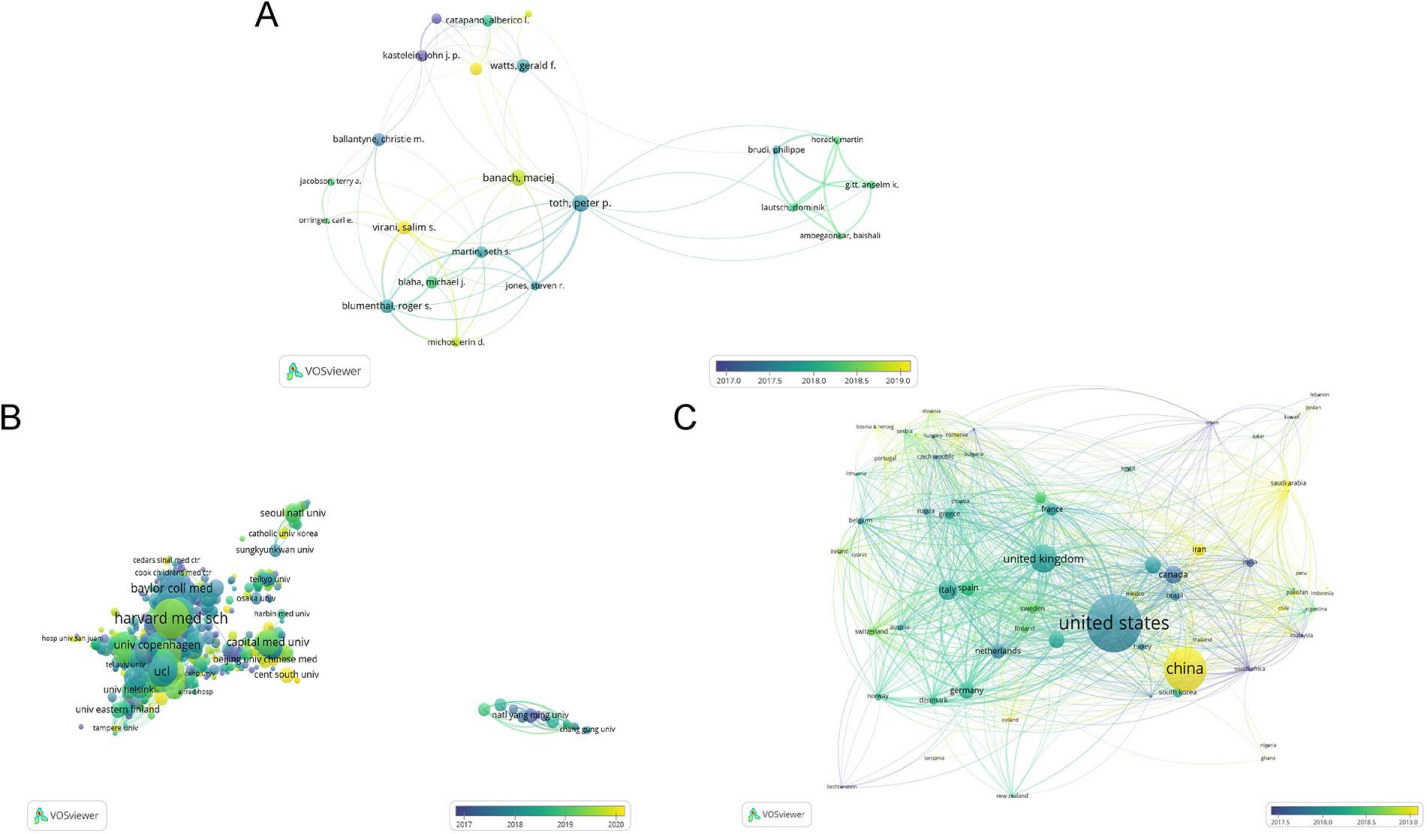
The analysis of co-authorship. **(A)** Network map of the quantity of published articles of authors related to CHD and lipid management research. **(B)** The analysis of authors’ institutions. **(C)** The analysis of authors’ countries. Different colors represent different clusters. The size of the circle is proportional to the number of publications.

As in Figure 3A, we analyze the top 50 authors with the highest number of publications in the field of CHD and lipid management from the data exported by Web of Science. The figure shows that there are varying degrees of collaboration among the top 50 authors. Figures 3B,C show partnerships across institutions and across countries, respectively. According to the size of the circles, in CHD and lipid management research, the authors are mainly from Harvard Medical School, Johns Hopkins University, and Baylor College of Medicine. According to F3C, authors in the field are mainly from the United States, China, and the United Kingdom. According to the colors, the United States has carried out research in this field earlier and has a large number of studies, while China has gradually increased its research in this field in recent years.

### 3.3 Citation analysis

#### 3.3.1 Author

**Figure 4:**
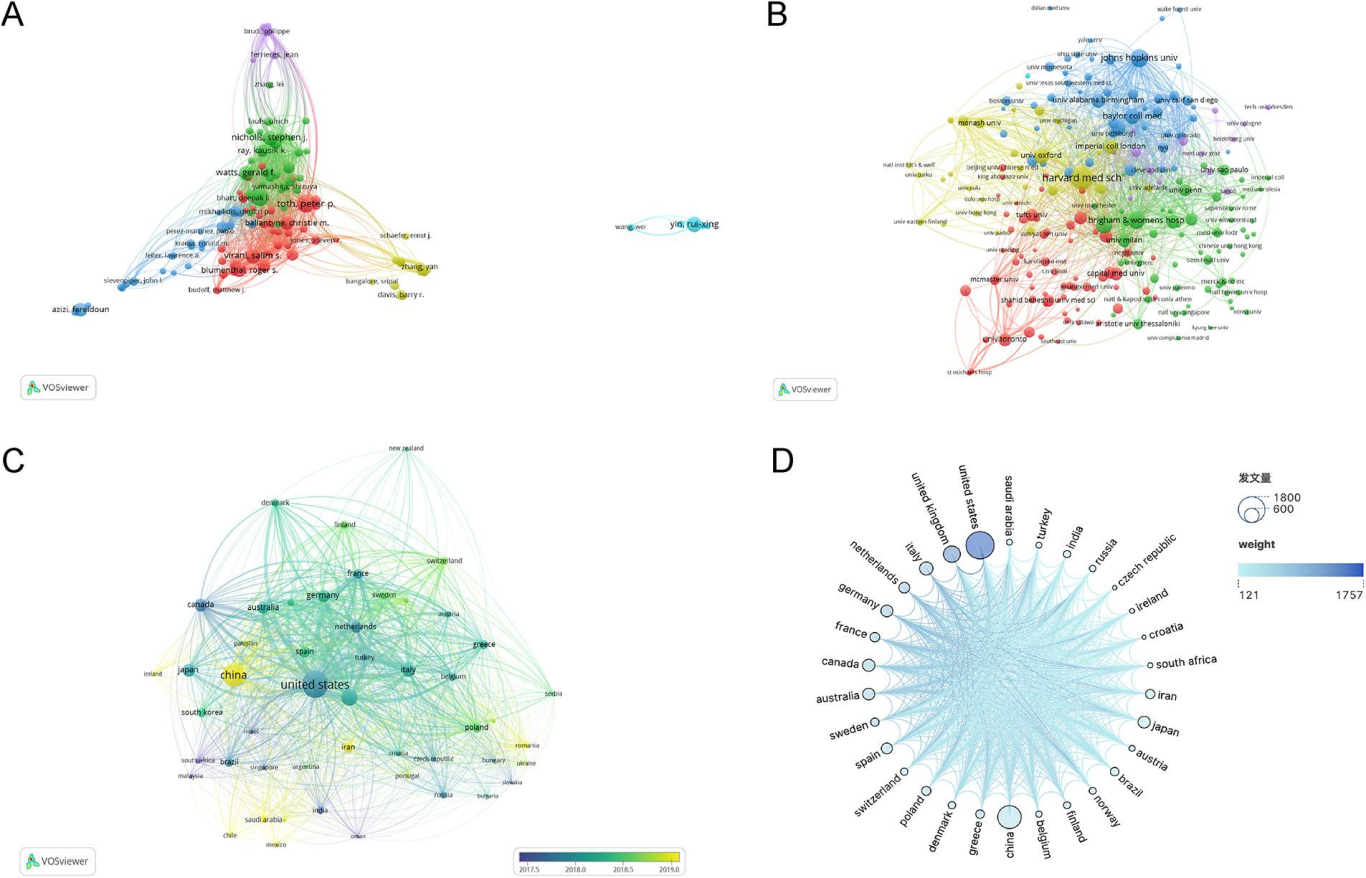
The analysis of citation. **(A)** Network map of authors related to CHD and lipid management research. **(B)** Distribution of organizations engaged in CHD and lipid management research. **(C)** Distribution of countries engaged in CHD and lipid management research. **(D)** Publications of different countries engaged in CHD and lipid management research.

By observing Table 1 and Figure 4A, we analyze the top 10 authors with the highest number of publications in the field of CHD and lipid management from the data exported by Web of Science. The top 3 authors, in order of the number of publications, are Maciej Banach (50), Toth, Peter P. (47) and Kausik Ray (46). Maciej Banach focuses on cardiovascular system and circulatory system. The research involves the influencing factors of cardiovascular disease (7, 8), lipid management (9–11) and other aspects.

**Table 1:** Top 10 yprolific authors of CHD and lipid management research.

Author	Documents	Organizition	h-index
Banach, Maciej	50	University of Western Australia	123
Toth, Peter P.	47	Johns Hopkins University	70
Ray, Kausik K.	46	Imperial College London	103
Catapano, Alberico L.	45	IRCCS Multimedica	107
Watt, Gerald	43	Brigham Young University	35
Nicholls, Stephen D.	40	Monash University	42
Ballantyne, Christie M.	39	Baylor College of Medicine	136
Yin, Rui-Xing	37	Guangxi Medical University	30
Kastelein, John J. P.	35	NewAmsterdam Pharm BVl	154
Blumenthal, Roger S.	35	Emory University	108

#### 3.3.2 Organization

As in Table 2 and Figure 4B, the top three institutions were the Harvard Medical School (137), Johns Hopkins University (98) and Brigham and Women's Hospital (91). The three most cited institutions were Harvard Medical School (14,830), Baylor College of Medicine (14,327) and Johns Hopkins University (13,873). It is worth noting that all the above institutions are located in the United States. It can be seen that the United States has rich research in this field and high recognition.

**Table 2:** Top 10 infuential institutions in research.

Organization	Decuments	Citations	Total link strength	Country
Harvard Medical School	137	14,830	3,146	United States
Johns Hopkins University	98	13,873	2,172	United States
Brigham and Women’s Hospital	91	11,748	2,277	United States
University College London	86	4,591	1,143	United Kingdom
Baylor College of Medicine	78	14,327	2,069	United States
University of Oxford	77	8,513	1,865	United Kingdom
Washington University in St. Louis	74	5,219	1,203	United States
Imperial College London	69	8,079	2,072	United Kingdom
The University of Alabama at Birmingham	68	3,136	607	United States
Università degli Studi di Milano	63	6,807	1,864	Italy

#### 3.3.3 Country

Figures 4C,D show the comparison of collaborations among different countries. A total of 111 countries have contributed to this field. According to the size of the circles and Table 3, the United States (1,802,19.22%), China (1,263,13.47%), and the United Kingdom (653,6.97%) published the largest number of papers and cooperated more closely with other institutions. From Figure 4C, it can be found that the publication years of most countries are mainly concentrated around 2018. The circles corresponding to China are lighter in color, indicating that related research in China has gradually increased in recent years. The corresponding circles for the United States and the United Kingdom are larger in area and darker in color, indicating that research in these countries started earlier and was more comprehensive. Figure 4D is a map of relations and cooperation between countries. It can be seen that the United States, as the country with the largest number of publications and citations in this field, has the most complex cooperative relationships, followed by the United Kingdom and Italy. Most countries have cooperative relations, which shows that the research in this field is relatively sufficient, and countries exchange and learn from each other to promote development.

**Table 3:** Top 10 countries in terms of publications.

Country	documents	citations	Total link strength	Percent (%)
United States	1,802	110,434	22,694	19.22%
China	1,263	21,887	7,110	13.47%
United Kingdom	653	53,087	14,132	6.97%
Italy	410	30,817	10,040	4.37%
Canada	349	27,267	6,324	3.72%
Germany	330	25,616	7,453	3.52%
Japan	329	14,206	2,815	3.51%
Australia	327	28,385	8,251	3.49%
Spain	281	13,600	4,766	3.00%
Netherlands	273	30,517	8,177	2.91%

### 3.4 Co-cited references analysis

**Figure 5:**
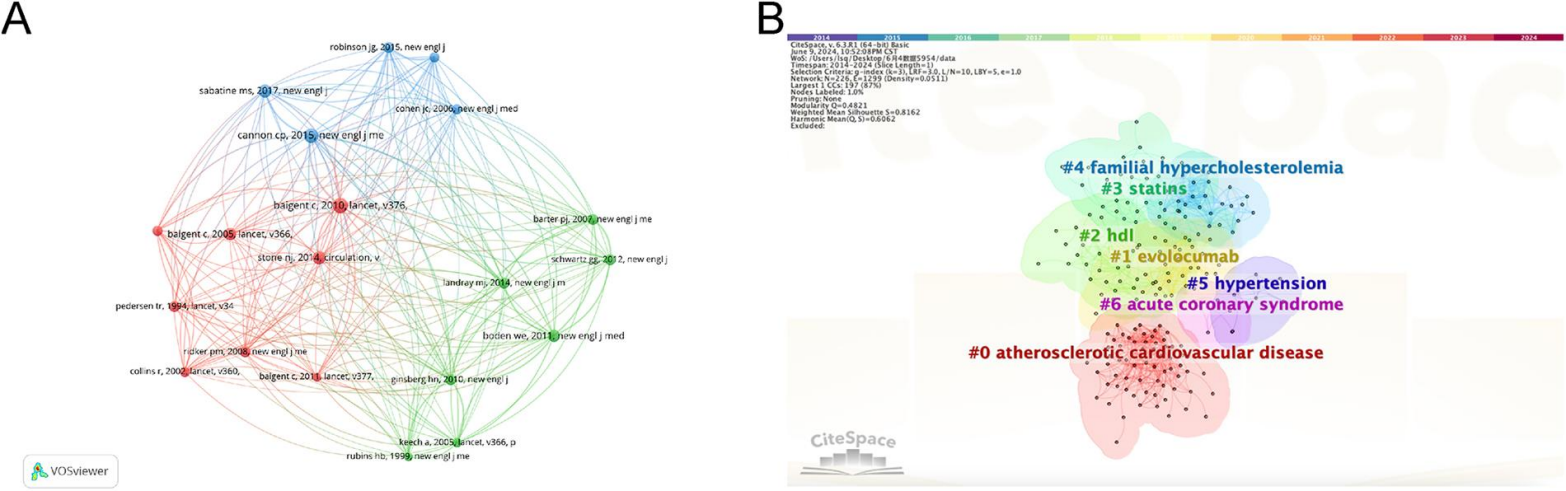
The analysis of co-cited references. **(A)** Top 20 co-cited references. **(B)** Cluster analysis of co-cited references.

Figure 5 shows the literature with a large number of co-citations. Through author analysis (Figure 5A) and cluster analysis (Figure 5B) of relevant highly cited literature, authors with greater influence and hot spots in this field have been identified. Combined with the data from Citespace's automated analysis (Table 4), the most cited papers are sourced from three prominent journals: The Lancet, The New England Journal of Medicine and Circulation.

**Table 4:** Top 10 author analysis diagram for highly cited references.

Number	Title	First author	Journal	Co-Citations	Citations	Year
1	Efficacy and safety of more intensive lowering of LDL cholesterol: a meta-analysis of data from 170,000 participants in 26 randomised trials	Baigent, Charlotte	Lancet	559	4,874	2010
2	Ezetimibe Added to Statin Therapy after Acute Coronary Syndromes	Cannon, Christopher P.	The New England Journal of Medicine	470	3,156	2015
3	2,013 ACC/AHA guideline on the treatment of blood cholesterol to reduce atherosclerotic cardiovascular risk in adults: a report of the American College of Cardiology/American Heart Association Task Force on Practice Guidelines	Stone, Neil J.	Circulation	395	4,282	2014
4	Evolocumab and Clinical Outcomes in Patients with Cardiovascular Disease	Sabatine, Marc S.	The New England Journal of Medicine	384	4,067	2017
5	Efficacy and safety of cholesterol-lowering treatment: prospective meta-analysis of data from 90,056 participants in 14 randomised trials of statins	Baigent, Charlotte	Lancet	345	5,452	2005
6	Niacin in patients with low HDL cholesterol levels receiving intensive statin therapy	Boden, William E.	The New England Journal of Medicine	329	2,096	2011
7	Third Report of the National Cholesterol Education Program (NCEP) Expert Panel on Detection, Evaluation, and Treatment of High Blood Cholesterol in Adults (Adult Treatment Panel III) Final Report	Gažarová, Martina	Circulation	317	12,631	2002
8	Estimation of the concentration of low-density lipoprotein cholesterol in plasma, without use of the preparative ultracentrifuge.	Friedewald, William T.	Circulation	295	4,600	1972
9	Rosuvastatin to prevent vascular events in men and women with elevated C-reactive protein	Ridker, Paul M.	The New England Journal of Medicine	294	5,241	2008
10	Effects of torcetrapib in patients at high risk for coronary events	Barter, Philip	The New England Journal of Medicine	279	2,547	2007

The three most co-cited articles in the field of lipid management of coronary heart disease were “Efficacy and safety of more intensive lowering of LDL cholesterol: a meta-analysis of data from 170,000 participants in 26 randomised trials” (493), “Ezetimibe Added to Statin Therapy after Acute Coronary Syndromes” (419) and “2013 ACC/AHA guideline on the treatment of blood cholesterol to reduce atherosclerotic cardiovascular risk in adults: a report of the American College of Cardiology/American Heart Association Task Force on Practice Guidelines” (359). Literature 1 was used to compare the prognosis of patients treated with different statin regimens by meta-analysis. The results showed that the incidence of cardiovascular events was lower with high-intensity statin therapy than with low-intensity statin therapy. In literature 2, an analysis of 18,144 patients hospitalized for acute coronary syndromes showed that the combination of simvastatin and ezetimibe was associated with lower cholesterol levels and a lower Kaplan–Meier event rate compared to simvastatin alone. Literature 3 is a guideline on lipid management, which mainly deals with specific strategies for lipid management.

Cluster labels were retained for only a fraction of the higher degree of association. As shown in F5B, clusters mainly included “#0 atherosclerotic cardiovascular disease”, “#1 evolocumab”, “#2 hdl”. “#3 statins”, "#4 familial hypercholesterolemia”, "#5 hypertension”, "#6 acute coronary syndrome”. The three largest clusters are #0, #1 and #2. It can be found that the research topics in the co-cited references mainly involved coronary heart disease related diseases and drugs.

### 3.5 Journals analysis

**Figure 6:**
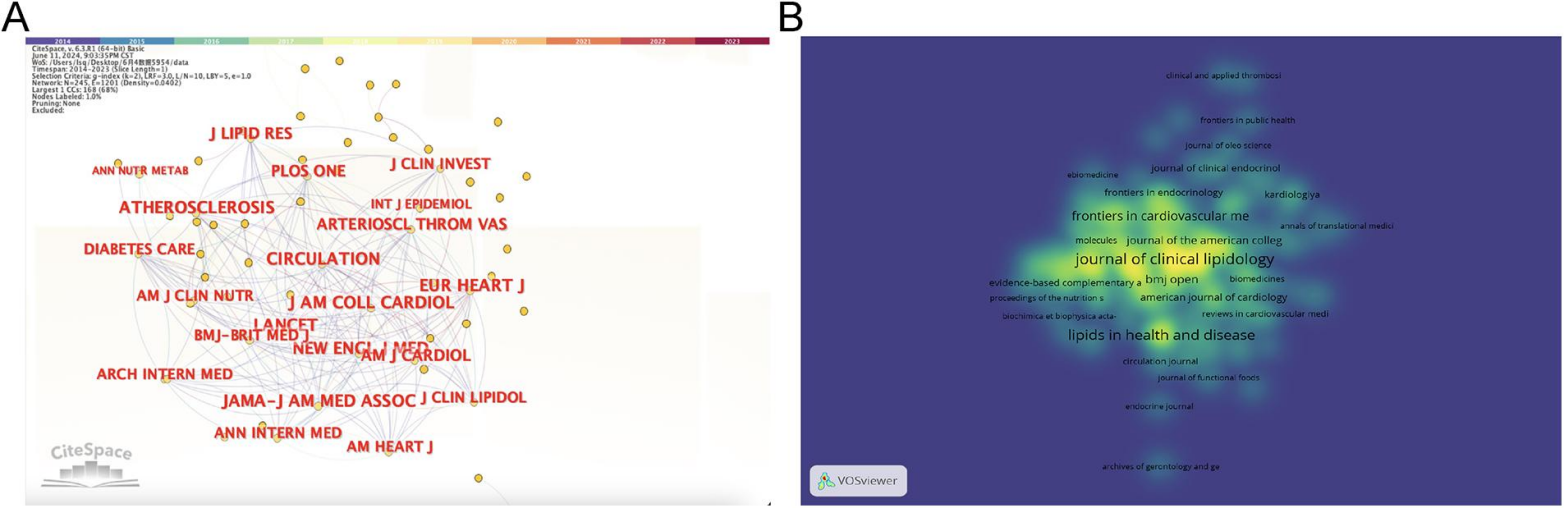
The analysis of journals. **(A)** Network map of journals related to CHD and lipid management research. **(B)** The density visualization map of journals related to CHD and lipid management research. The higher the density, the closer it is to red; the lower the density, the closer it is to blue.

Figure 6A shows the 22 journals with the most published articles in the field. According to Table 5 and Figure 6B, the main sources of literature in this field are Journal of Clinical Lipidology (160), Atherosclerosis (132) and Lipids in Health and Disease (132). The most cited journals were Atherosclerosis (7,915), Journal of clinical lipidology (5,427) and Nutrients (3,354). It can be seen that Journal of clinical lipidology and Atherosclerosis have high recognition in this field.

**Table 5:** The top 10 journals of origin of articles on CHD and lipid management research.

Journal	Documents	Citations	Total link strength	IF (2023)	Category (JCR)
Journal of clinical lipidology	160	5,427	829	3.6	Pharmacology& Pharmacy(Q2)
Atherosclerosis	132	7,915	1,005	4.9	Cardiac& Cardiovascular Systems(Q1)
Lipids in health and disease	132	2,623	287	3.9	Biochemistry& Molecular Biology(Q2)
Nutrients	100	3,354	275	4.8	Nutrition & Dietetics(Q1)
Plos one	98	2,243	143	2.9	Multidisciplinary Sciences(Q1)
Journal of the American heart association	89	2,939	307	5	Cardiac& Cardiovascular Systems(Q1)
Frontiers in cardiovascular medicine	64	564	258	2.8	Cardiac& Cardiovascular Systems(Q2)
Bmj open	61	846	140	2.4	Medicine,General&Internal(Q1)
Cardiovascular diabetology	57	2,617	153	8.5	Cardiac& Cardiovascular Systems(Q1)
Journal of atherosclerosis and thrombosis	57	816	136	3	Peripheral Vascular Disease(Q2)

The impact factors (IF) of the top 10 journals with the most publications in this field ranged from 2.4 to 8.5, with a certain gap in influence. 25% of the top 10 journals were in the Cardiac& Cardiovascular Systems category. Journal Citation Reports (JCR) classifies the impact factors of journals into quartiles (Q1–Q4), of which Q1 is the highest.60% of the top 10 journals belong to Q1% and 40% belong to Q2, which shows that the research in this field has a certain frontier and importance.

### 3.6 Keyword analysis

**Figure 7:**
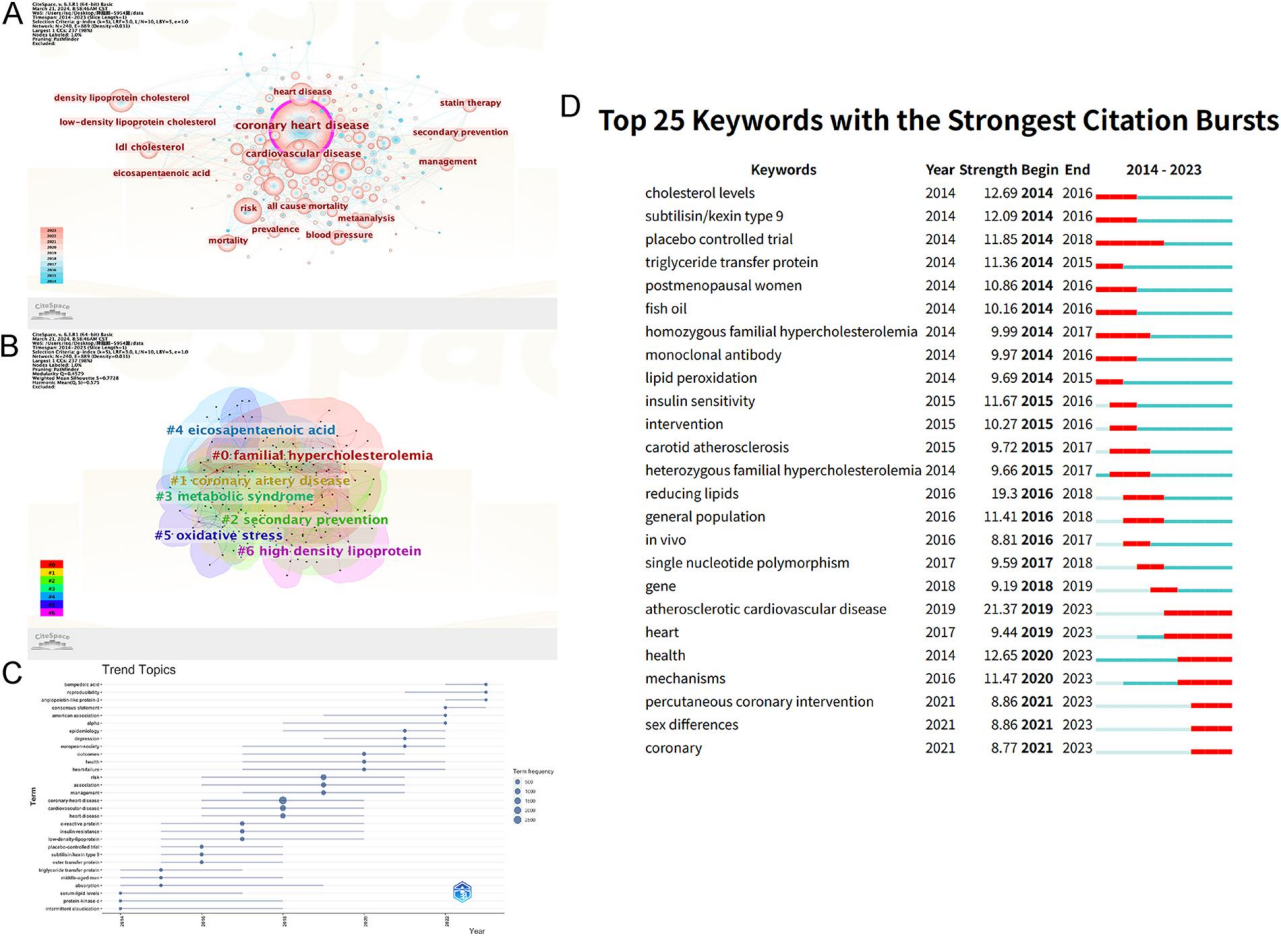
The analysis of keywords. **(A)** High-frequency keywords and its relationship in CHD and lipid management research. **(B)** Keywords cluster analysis co-occurrence map. **(C)** Keyword time zone map. **(D)** Top 25 keywords with the strongest citation bursts. The size of the circle was positively correlated with attention.

Keyword analysis is a valuable approach to uncovering the fundamental content of literature, comprehending the developmental trajectory, identifying research focal points, gauging emerging trends, and predicting the future course of the subject matter. In this study, we focused on the “keyword” node and conducted a visual analysis of keyword co-occurrence as illustrated in Figure 7A. We conducted an analysis of the top 10 high-frequency keywords, which were highly relevant to the topic, and presented the results in Table 6. Notably, the three most frequently occurring keywords were “coronary heart disease” (3,388), “cardiovascular disease” (1,649), and “risk” (1,018). Using “coronary heart disease” as search term is normal, as it is a frequent keyword in search results. The risk factors of coronary heart disease have received extensive attention.

**Table 6:** Top 10 high-frequency keywords and centrality indices in CHD and lipid management research.

NO.10	Centrality	Count	Keyword
1	0.27	3,388	coronary heart disease
2	0.07	1,649	cardiovascular disease
3	0.05	1,018	risk
4	0.03	794	myocardial infarction
5	0.06	758	heart disease
6	0.07	745	density lipoprotein cholesterol
7	0.02	743	coronary artery deiease
8	0.02	694	risk factors
9	0.02	543	association
10	0.04	495	cholesterol

The LLR algorithm was employed to conduct cluster analysis of the keywords. In Figure 7B, we present the clustering of 7 keywords in WOS, which includes “#0 familial hypercholesterolemia”, "#1 coronary artery disease”, “#2 secondary prevention”, "#3 metabolic syndrome”, "#4 eicosapentaenoic acid”, "#5 oxidative stress” and “#6 high density lipoprotein”. Cluster #0, #2 can be classified as related diseases; cluster #1, #3 can be classified as treatments; cluster #5, #6 can be classified as mechanisms.

According to the Figure 7C, trend topics change over time. From 2014 to 2020, the research hotspots were american association, consensus statements, epidemiology, apoptosis, c-reactive protein, ester transfer protein, protein-kinase-c, triglyceride transfer protein and so on. Researchers have focused on the guidelines, epidemiology, and mechanisms of lipid management in CHD. The new lipid-lowering drug bempedoic acid, lymphocyte ratio and glucose homeostasis have been the research hotspots in the past 1–2 years. Research trends are gradually shifting toward new therapeutic drugs and more specific pathogenesis.

## 4 Discussion

By utilizing cluster analysis and burst keyword analysis, we are able to provide an overview of the research hotspots during various time periods and show how this field of study has developed. Next, we explored the lipid management research horizon and influencing factors for CHD. The discussion section focuses on the aforementioned analytical results and lipid management frontiers.

### 4.1 Regular results

Based on the analysis of cooperation between countries and institutions, we can analyze the research and development trends in this field. From 2014 to 2023, the number of publications declined but still maintain a high level. The number of citations increased year by year, indicating that the lipid management of CHD also plays an important role in other fields, which may be reflected in the prevention and treatment of some comorbidities. Although more and more countries and institutions are conducting research in this field, most papers are still produced by a small number of countries and institutions. The top 10 countries accounted for more than 90% of the total number of publications. the United States (1,802), China (1,263), and the United Kingdom (653) were the three countries with the largest number of articles, accounting for 39.7% of the total number of papers in this field. It can be seen that the research in these three countries has made great contributions to the development of this field. Among them, the number of publications and citations of the United States were much higher than those of other countries, and it cooperative relationships has the most abundant. It can be seen that the United States has the most research in this field and is highly recognized.

Harvard Medical School (137), Johns Hopkins University (98) and Brigham and Women's Hospital (91) were the three institutions with the most published articles, all from the United States. Of the top 10 institutions, 6 are from the United States, 3 are from the United Kingdom and 1 is from Italy. This suggests an imbalance in the distribution of academic resources in this field. The United States may have more top researchers, institutions and resources. The research in the United States covers a wide range of topics, including clinical research, molecular and cellular research, data analysis, and new drug development.

Maciej Banach, as the author with the highest number of publications, has great influence in this field. He published 1,226 articles in total, including “Obicetrapib on top of maximally tolerated lipid-modifying therapies in participants with or at high risk for atherosclerotic cardiovascular disease: rationale and designs of BROADWAY and BROOKLYN”,"Recommendations of the Experts of the Polish Cardiac Society (PCS) and the Polish Lipid Association (PoLA) on the diagnosis and management of elevated lipoprotein(a) levels”, “The Progress and Research Trends of Statin Medications: The Advanced Epidemiological and Bibliometrical Assessment” and so on. His research in this field mainly focuses on the related drugs for lipid management and lipid metabolism disorders.

The top 10 journals with the most publications and citations were renowned high-quality journals, which confirming the research significance of lipid management in CHD. The top three journals with the most published articles were Journal of Clinical Lipidology (160), Atherosclerosis (132) and Lipid in Health and Disease (132). Among them, Atherosclerosis ranked second in terms of the number of publications, but had the highest citations (7,915), indicating that the journal had a high degree of attention and recognition in this field.

### 4.2 Influencing factors and lipid management of CHD based on cluster analysis

Potential patterns and categories within a dataset can be identified by cluster analysis, which aids in comprehending the properties and structure of the data. The terms “familial hypercholesterolemia,” “coronary artery disease,” “secondary prevention,” “metabolic syndrome,” “eicosapentaenoic acid,” “oxidative stress,” and “high-density lipoprotein” are clustered together in the context of CHD and lipid management, which can be categorized into three aspects: related diseases, treatment, and mechanisms.

**Figure 8:**
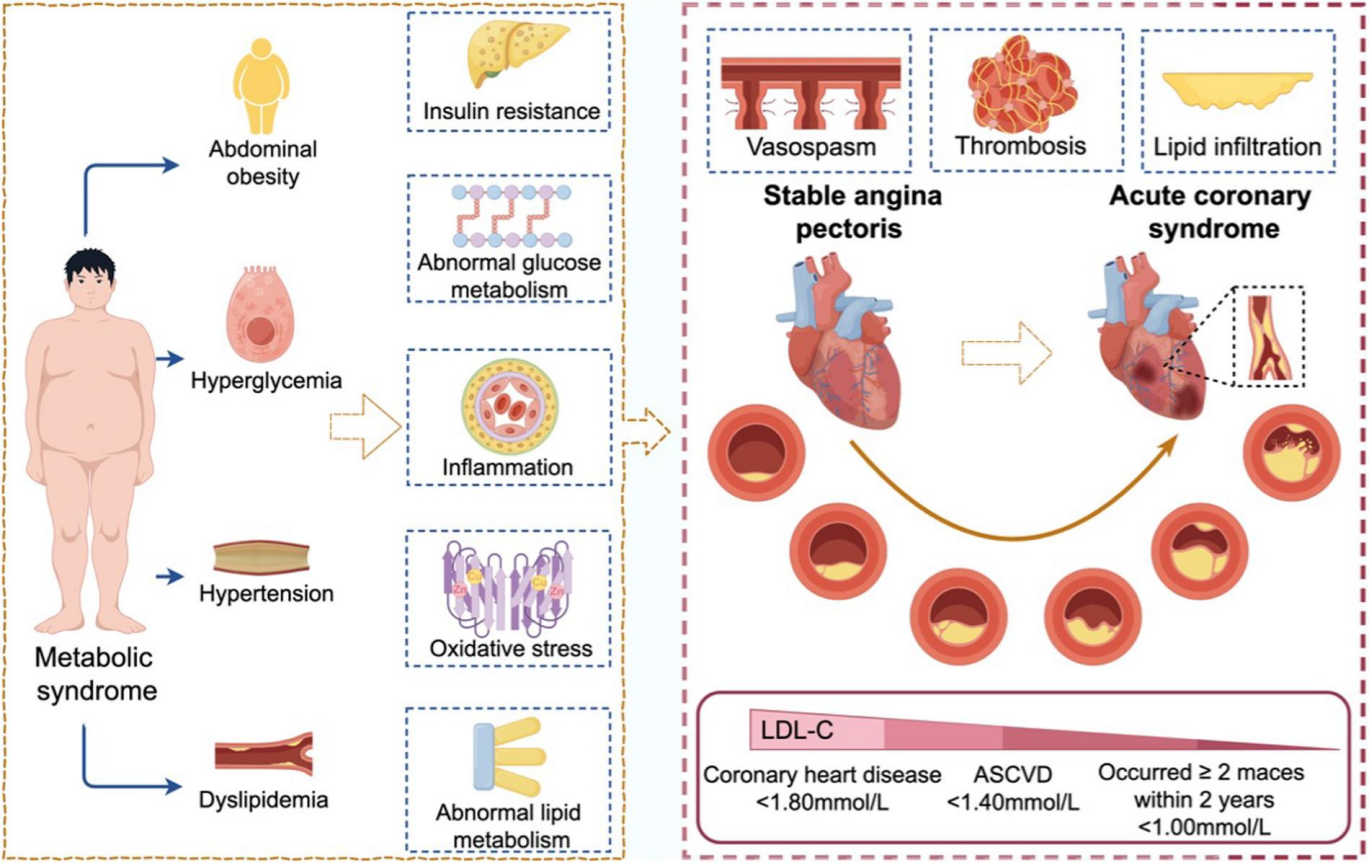
The mechanism of coronary heart disease and the objective of lipid management. (Figure 8 is created on figdraw.com and have been authorized for use. Caption: As shown in Figure 8, metabolic syndrome is a clinical syndrome characterized by the clustering of obesity, hyperglycemia, dyslipidemia, and hypertension. The main pathological mechanisms in patients with metabolic syndrome include insulin resistance, abnormal glucose metabolism, inflammatory response, oxidative stress, and abnormal lipid metabolism, which can promote lipid infiltration and thrombus formation. After developing into CHD, if lipid infiltration, vasospasm, and thrombus formation further worsen, it may progress to acute coronary syndrome (ACS). According to the different progressions of CHD, the control targets for LDL-C are below 1.8 mmol/L, 1.4 mmol/L, and 1.0 mmol/L, respectively).

According to Figure 7B, metabolic syndrome is a significant risk factor for CHD. As illustrated in Figure 8, metabolic syndrome may increase the incidence of CHD through mechanisms such as abnormal glucose metabolism, insulin resistance, inflammatory response, oxidative stress, and aberrant lipid metabolism. Effective lipid management helps to inhibit the future development of CHD. The lipid management of metabolic syndrome can be divided into pharmacological and non-pharmacological treatments. Pharmacological treatment is primarily based on statins. In addition, fibrates can effectively restore the lipid levels (mainly affecting triglycerides and HDL-cholesterol) in patients with metabolic syndrome and improve insulin resistance (12–16). Non-pharmacological treatment mainly focuses on lifestyle interventions, which are the foundation of lipid-lowering therapy. It mainly includes weight control, exercise, and a balanced diet (17–19), which can effectively improve lipid levels in patients with metabolic syndrome, reduce oxidative stress levels, and thereby lower the incidence of CHD (20, 21).

Oral medication is the most widely applied method in lipid management. The classes of oral lipid-lowering medications include statins, fibrates, niacin, bile acid sequestrants and cholesterol absorption inhibitors. For now, statins continue to be the mainstay of clinical treatment. Statins mainly function by blocking the cholesterol synthesis enzyme HMG-CoA reductase to reduce LDL-C in the plasma (22), thereby lowering LDL-C levels and helping to prevent atherosclerosis. Statins also have immune-regulation and anti-inflammatory effects. Recent studies have shown that statins can effectively avoid significant adverse cardiovascular events in hemodialysis patients after PCI (23) and to improve cardiac sympathetic nerve activity in patients following reperfusion therapy for the first STEMI (24). Pretreatment with high-intensity statins can greatly enhance post-PCI outcomes (25).

The statin-based lipid management has been widely applied in the treatment of patients with CHD. Statin combination therapies overcome monotherapy limitations in meeting complex lipid management requirements of CHD patients. It is required to introduce non-statin lipid-lowering therapy for patients who are intolerant to statins or who have not reached the recommended LDL-C target despite taking statins at the maximum tolerable dose.

The objectives for decreasing cholesterol also alter if the condition is ACS. When treating ACS, statins, PCSK9 inhibitors, and ezetimibe (26) have good safety and effectiveness profiles. Previous clinical data have mostly focused on statins for the control of lipids. The lipid-lowering target is LDL-C <1.40 mmol/L, with an LDL-C reduction target of >50% from baseline.

According to the recommended stepped lipid-lowering management, ezetimibe should be administered together if patients do not achieve LDL-C <1.40 mmol/L within 4–6 weeks. A PCSK9 inhibitor should be added to patient's high-intensity combination therapy if their LDL-C level is still above this target (27). The lipid-lowering target should be lowered to LDL-C <1.00 mmol/L if two or more major adverse cardiovascular events occur within a two-year period.

Oxidative stress impacts CHD mainly by inducing damage to coronary artery endothelial cells, triggering inflammatory responses, and stimulating the growth of vascular smooth muscle cells and the production of extracellular matrix. New research has found a correlation between fatty acids and oxidative-antioxidant imbalances. In patients with coronary atherosclerosis, levels of polyunsaturated fatty acids such as eicosapentaenoic acid and linoleic acid are significantly reduced, oxidative stress levels increase by 17%, and serum total antioxidant defense decreases by 45%. This implies that notable alterations in the fatty acid profile are associated with modifications in the state of oxidative-antioxidant status (28).

### 4.3 Risk factors and intervention of the lipid management of CHD based on burst keywords analysis

In terms of epidemiology, clinical presentation, diagnostic testing and treatment, women and men differ significantly (29). Women are always less likely to develop CHD than men at any age, but this difference gradually closes as age increases (30). Moreover, even after menopause, the progression rate of coronary atherosclerosis in women remains slower than in men (31). Women have unique plaque characteristics: smaller plaques, less calcification, and less obstructive. Moreover, only in women is there a close correlation between mental stress and endothelial dysfunction (32). Recent studies have shown that although men have a higher coronary artery calcium (CAC) burden, women have a higher likelihood of plaque erosion in non-calcified plaques, which is mainly reflected in acute and long-term cardiovascular mortality (33, 34). Especially for postmenopausal women, there is often an increase in the risk of cardiovascular diseases, which is related to the vascular protective effect of estrogen. The occurrence and exacerbation of CHD in postmenopausal women may exert an influence by affecting lipid metabolism, microcirculation, endothelial cell function, inflammatory response, genetic polymorphism and other aspects (35–40). In terms of lipid management, the role of estrogen is reflected in the fact that women's age-standardized rate of lipid-lowering drug use is lower than men’, and women start taking lipid-lowering medications later in life.

The onset and progression of CHD are significantly influenced by genetic factors. There is some familial aggregation of CHD, meaning that an individual's chance of getting the disease is often higher if there is a family history of the condition. Single nucleotide polymorphisms (SNPs) are widely utilized method in genetic research these days. SNPs can lead to an increased risk of CHD. Research on the effect of SNPs at various gene loci on the risk of CHD has been dominated in recent years. The SNP at the rs7014968 locus of the XKR6 gene may increase the risk of CHD by raising serum total cholesterol (TC) levels (41). The blood cholesterol levels and carotid intima-media thickness are linked to the rs1883025 locus of the ABCA1 gene and the rs881844 locus of the STARD3 gene, which increases the susceptibility to CHD (42). The latest research suggests that polymorphisms in the cholesteryl ester transfer protein (CETP) gene may significantly affect cardiovascular risk, although the precise mechanisms are yet unknown.

Familial hypercholesterolemia (FH) includes heterozygous familial hypercholesterolemia (HeFH) and homozygous familial hypercholesterolemia (HoFH). In comparison to HoFH, HeFH is more prevalent, has a later onset of the illness, develops the illness later, and responses better to medication. HeFH can lead to premature CHD. It used to be considered that the abnormality in lipids is mainly reflected in the elevated levels of LDL. However, recent studies have found that HDL particles in patients with HeFH show abnormalities, including changes in composition, reduced ability to promote cholesterol efflux from macrophages, and impaired anti-inflammatory and antioxidant activities, suggesting that HDL dysfunction may also be one of the mechanisms by which patients with HeFH have a high cardiovascular risk (43). Medication therapy alone is not always sufficient to achieve treatment objectives for pediatric patients. Therapeutic apheresis, which selectively removes lipoproteins from the blood, is mostly used for adult treatment, and may be considered for treatment after tailoring the treatment plan according to the child's specific situation (44).

Lipid concentrations are regulated in part by PCSK9. According to experimental findings, PCSK9 and LDL-C exhibit a favorable correlation in patients who have not received lipid-lowering therapy (45). PCSK9 accelerates the progression of atherosclerotic cardiovascular diseases through multiple pathways, including the control of lipid metabolism, immune responses promotion, platelet activation enhancement, thrombus formation promotion, induction of cell apoptosis, facilitation of pyroptosis and autophagy inhibition. A Mendelian randomization study identified drug targets (46) by their association with lipids (HDL-C, LDL-C, and triglycerides), among which PCSK9 is a target that has beneficial effects in the prevention or cure of CHD. PCSK9 remains an effective therapeutic target for patients who are intolerant to statins or who have not achieved lipid targets after maximum dose statin therapy (47). Furthermore, targeting synthetic transcription factors of PCSK9 and its downstream molecules is still crucial for the development of novel lipid-lowering drugs because PCSK9 can mediate the degradation of LDLR through both extracellular and intracellular pathways, resulting in hyperlipidemia. This provides a dependable path for future study on CHD patients’ lipid management therapies (48).

Lipid peroxidation serves as a pivotal pathological mechanism in CHD, with its metabolites demonstrating potential as diagnostic and staging biomarkers (49). Besides, serum levels of HDLox were significantly increased in CHD patients, suggesting its diagnostic value (50). Lipid peroxidation and oxidative stress are pathologically intertwined (51). Disturbed blood flow during the early phases of atherosclerotic plaque formation increases the permeability of endothelial cells to LDL and produces more ROS, which accelerates the oxidation of LDL. HDL exerts atheroprotective effects primarily through facilitating reverse cholesterol transport from foam cells and mediating antioxidant activity. However, its function may be damaged by ROS. Specifically, ROS can directly oxidize and modify amino acids in HDL, or indirectly modify HDL proteins by producing reactive aldehydes from lipid peroxide fragments (LOOH) (52). Nevertheless, HDL is still effective in clearing the accumulation of LOOH in LDL and neutralizing ROS, thereby reducing oxidative damage and slowing disease progression (53).

Figure 7D claims that for the last 10 years, the research hotspot has been continuous changing. In 2014, cholesterol levels were under the spotlight, and during this period, the application of monoclonal antibodies was the most popular therapeutic strategy. PCSK9 inhibitors are a class of monoclonal antibodies, which can directly inhibit the binding of PCSK9 to the low-density lipoprotein receptor (LDLR), thereby promoting the transport of LDL-C by LDLR. It has been shown that PCSK9 inhibitors have been demonstrated in clinical trials to dramatically change the lipid makeup of plasma and lipoprotein particles (54). It has also been observed that psoriasis patients treated with IL-17A monoclonal antibodies have restored normalized lipid metabolism (55), indicating that other monoclonal antibodies could potentially be therapeutic medications for cardiovascular illnesses. Furthermore, inhibiting the mRNA-dependent synthesis pathway can also impede the production of PCSK9. One example is inclisiran, a small interfering RNA therapeutic administered subcutaneously at months 0 and 3, then every 6 months, achieving sustained LDL-C reduction (56).

Fish oil garnered significant attention at the same time. Unsaturated fatty acids (ω-3) found in fish oil, such as docosahexaenoic acid (DHA) and eicosapentaenoic acid (EPA), have metabolic and cardiovascular regulating effects and are frequently used as preventative medication for cardiovascular disorders (57–59).The American Heart Association (AHA) proposed the use of fish oil as primary prevention of CVD in 2017 (60). Studies show that EPA and DHA can favorably regulate a number of established CVD risk factors, such as lipid modulation, which decreases the incidence of CHD (61). Supplemental fish oil lowers the prevalence of CHD in persons with prediabetes and diabetes (62). Patients with cardiometabolic illness who take fish oil have longer life expectancies and significantly smaller chances of cardiovascular and all-cause mortality (63).

Treatment for CHD often involves percutaneous coronary intervention (PCI). The post-operative residual hazards may be associated with the systemic pro-atherosclerotic effects of residual LDL-C. Priority should be given to statin medications for all CHD patients undergoing PCI surgery, and non-statin lipid-lowering medications should be added as necessary to meet the recommendations’ suggested LDL-C treatment targets (64). Research has demonstrated that in individuals with acute coronary syndrome, early beginning of statin medication therapy can successfully reduce long-term cardiovascular events following PCI. In addition, the use of combination medications in lipid-lowering regimens has been extensively promoted (65) possibly due to their superior ability to lower LDL-C levels in patients following PCI (66).

Lifestyle modification is very important in the preventing and treating CHD, especially among young people with high genetic risk, who may benefit more from improving lifestyle to prevent cardiovascular disease than other genetic subgroups (67). Lifestyle interventions include adopting a low-fat diet, weight management, regular exercise, smoking cessation, and moderating alcohol consumption and other unhealthy lifestyle changes. Among these, dietary management is one of the core components of CHD management. Epidemiological studies have shown that unhealthy dietary habits increase the risk of CHD (68, 69). Therefore, dietary intervention plays a crucial role in the Long-term low-fat diet (LFD) can significantly reduce waist circumference and body mass index in patients with metabolic syndrome (70). At the molecular level, LFD can improve the shift of blood fatty acid composition in CHD patients in a healthier direction (71). It also significantly reduces the size of the major LDL fraction, the susceptibility of LDL to oxidation, plasma oxidized LDL concentration (72), as well as total cholesterol levels and C-reactive protein content. Reducing the risk of cardiovascular disease by ameliorating dyslipidemia and inflammation levels (73, 74). A low-salt diet is also strongly associated with a reduced risk of atherosclerosis and has a synergistic blood-pressure-lowering effect. Among three antihypertensive regimens of losartan monotherapy, losartan/hydrochlorothiazide and irbesartan/hydrochlorothiazide combination therapy, patients with low-salt diet have a more significant reduction in blood pressure (75).

In addition, exercise has been shown to improve left ventricular ejection fraction, reduce resting heart rate, and lower the risk of angina, arrhythmia, and coronary restenosis in patients with CHD after PCI (76, 77). Exercise also has positive effects on cardiopulmonary function, blood pressure, and lipid levels (78). Notably, compared to either LFD or moderate-intensity aerobic exercise training (MIAET) alone, the combination of LFD and MIAET exercise for 10 weeks demonstrated superior effects on lipid control in obese patients with dyslipidemia. Specifically, TG, T-Ch, LDL and HDL were significantly improved (73). Patients’ TG and TC also improved after cardiac rehabilitation (79). However, the reality is that patients with CHD often have inadequate adherence to lifestyle changes after discharge. Only 29% of patients were able to achieve the expected goals in all three aspects: weight loss, smoking cessation and increasing physical activities (80). In the era of digital health, the combining of modern internet technology and secondary prevention of CHD may open up a new way to improve the compliance of patients to modifying unhealthy lifestyle habits (81).

## 5 Conclusions

This article analyzes the current state and trends of research on lipid management of CHD from 2014 to 2023 through bibliometric research. Gender, genetics, PCSK9 targets, and lipid peroxidation are the risk factors affecting CHD that are linked to lipid management. Research foci in recent years have primarily been on novel medication development, including Bempedoic acid and Inclisiran, the molecular effects of related medicines, and targeted therapeutic approaches for lipid management in CHD. The goals and targets for lipid management in CHD are still primarily based on guidelines. It is necessary to evaluate the risks of CHD patients, construct related lipid-lowering targets based on risks, and establish corresponding lipid management programs. One prerequisite for plaque regression is consistent and efficient lipid management. In recent years, researches of plaque reverse continue to increasing, effective lipid management contribute to achieving this goal. Future research hotspots may still focus on more precise treatment protocols and the exploration of novel mechanisms. Due to the methodological limitations inherent in this type of research, our study primarily relied on English-language literature, potentially overlooking non-English literature that may contribute to the research findings. Additionally, there was a temporal gap between the publication period of the articles cited and the time of writing this paper, which resulted in the exclusion of more recently published studies. However, our research team remains committed to actively tracking the latest advancements in related fields and will promptly update our findings as new breakthroughs emerge.

## Data Availability

The original contributions presented in the study are included in the article/supplementary material, further inquiries can be directed to the corresponding author/s.
